# Current Advances and Future Prospects in the Use of a Low-Carbohydrate Diet in Managing People with Type 2 Diabetes: A Systematic Review of Randomised Controlled Trials

**DOI:** 10.3390/ijerph22091352

**Published:** 2025-08-28

**Authors:** Omorogieva Ojo, Osarhumwese Osaretin Ojo, Yemi Onilude, Victoria Apau, Ivy Kazangarare, Tajudeen Arogundade, Joanne Brooke

**Affiliations:** 1 School of Health Sciences, Faculty of Education, Health and Human Sciences, University of Greenwich, Avery Hill Campus, London SE9 2UG, UK; o.onilude@greenwich.ac.uk (Y.O.); v.apau@greenwich.ac.uk (V.A.); i.kazangarare@greenwich.ac.uk (I.K.); t.arogundade@greenwich.ac.uk (T.A.); 2 South London and Maudsley National Health Service (NHS) Foundation Trust, Denmark Hill, London SE5 8AZ, UK; osarhumwese.ojo@slam.nhs.uk; 3 Centre of Social Care, Health, and Related Research, Birmingham City University, Westbourne Rd, Birmingham B15 3TN, UK; joanne.brooke@bcu.ac.uk

**Keywords:** type 2 diabetes, low-carbohydrate diets, glycaemic control, body mass index, dietary intervention

## Abstract

Background: There is a worldwide increase in the prevalence of type 2 diabetes, and strategies for managing this condition include dietary interventions. These interventions include the use of a low-glycaemic index diet, high-fibre and prebiotic diets, and low-carbohydrate diets (LCDs), which improve glycaemic control, reduce the risk of diabetic complications, and promote health. However, the definition of LCDs varies across the literature, and the use of LCDs in managing people with diabetes is often seen as controversial. Therefore, the aim of this review is to examine current advances and future prospects in the use of LCDs in managing people with type 2 diabetes. Method: A systematic review of randomised controlled trials, which applied both the PRISMA and PICOS frameworks. Databases including MEDLINE, APA PsycInfo, Academic Search Premier, CINAHL Plus with Full Text, APA PsycArticles, and Psychology and Behavioural Sciences Collection were searched through EBSCOHost. The EMBASE database and reference list of articles were also searched for articles of interest. Two researchers conducted the searches independently from database inception to 28 August 2025. However, based on the inclusion criteria, the year of publication of studies was restricted to articles published from 2021. The search terms were combined using Boolean operators (AND/OR), and duplicates were removed in EndNote. The articles were screened for eligibility based on inclusion and exclusion criteria by two researchers. Results: The findings identified that an LCD is significantly (*p* < 0.05) more effective in reducing glycaemic parameters compared to a usual diet, standard care, or a control diet in people with type 2 diabetes. Similarly, the effect of LCD was significant (*p* < 0.05) in reducing BMI in patients with type 2 diabetes compared with the control diet. However, an LCD did not appear to have a significant (*p* > 0.05) effect on lipid parameters compared to a control diet. Conclusion: This systematic review found that LCDs are significantly (*p* < 0.05) more effective in promoting glycaemic control than a usual diet, standard care, or a control diet in people with type 2 diabetes. In addition, LCDs can be an effective strategy for reducing BMI in individuals with type 2 diabetes, particularly when implemented as part of a structured, sustained dietary intervention. However, there was variability in the findings of the studies included with respect to glycaemic control and BMI. Furthermore, the impact of LCD on glycaemic control did not appear sustainable in the long term. LCDs did not have a significant (*p* > 0.05) effect on lipid parameters compared to a control diet.

## 1. Introduction

The prevalence of type 2 diabetes is on the increase globally, both in developed and developing economies [1,2]. The impact of type 2 diabetes, if undiagnosed, can be profound, including the development of acute complications such as hyperglycaemia and hyperosmolar hyperglycaemic state. In poorly managed people with type 2 diabetes, chronic complications include cardiovascular diseases, diabetic osteopathy, neuropathy, nephropathy, retinopathy, and diabetic foot ulcers [3]. Risk of diagnosis of type 2 diabetes includes a genetic predisposition and lifestyle factors, such as a lack of physical activity and poor dietary intake. In this regard, dietary interventions are one of the strategies for managing people with type 2 diabetes [2]. In particular, the use of low-glycaemic index diet, high fibre and prebiotic diets, and low-carbohydrate diets (LCDs), all of which have been recommended for improving glycaemic control, reducing the risk of diabetic complications, and promoting overall health [4,5,6].

### 1.1. Low-Carbohydrate Diets

The recommended amount of carbohydrate intake is about half (45 to 65%) of a person’s energy requirement [7]. Furthermore, the current UK government advice on carbohydrate intake for the general population is based on the recommendations of the Scientific Advisory Committee on Nutrition, which is 50% of total dietary energy should be obtained from carbohydrates [8]. The recommendations also identify that carbohydrate intake should be obtained mainly from starchy foods, which include high fibre or whole grain foods where possible [8]. However, there is evidence that LCDs improve glycaemic control in people with type 2 diabetes [6].

Dening et al. [4] defined LCDs as 10% to <26% carbohydrate of total energy intake, which promotes significant clinical outcomes in people with type 2 diabetes. On the other hand, Ren et al. [5] referred to LCDs as a dietary strategy that reduces the energy supply ratio of carbohydrates and increases the energy supply ratio of fats and protein. In this regard, various categories of carbohydrate diets have been defined, depending on the amount of energy they contribute daily [8,9].

According to Dening et al. [4], very LCDs contribute <10% carbohydrates of total energy or <50 g/day carbohydrates, while LCDs contribute 10 to <26% of total energy or 50–129 g/day carbohydrates [9]. Furthermore, moderate carbohydrate diets provide 26–45% of total energy or 130–225 g/day carbohydrates, while high-carbohydrate diets supply >45% of total energy intake or >225 g/day carbohydrates [9].

Dening et al. [9] proposed the Carb-Cal Model for standardising the level of carbohydrate intake that is adequate. Based on the fact that there are 4 calories per gram of carbohydrates, the level of carbohydrates being prescribed can be calculated using a mathematical equation: Total calories per day, multiplied by percentage of total energy obtained from carbohydrates, divided by 4. Therefore, an individual consuming 2000 calories/day and on LCD should have 200 to <520 calories obtained from carbohydrates, which would be 50–<130 g/day of carbohydrates [9].

### 1.2. Why This Review Is Important

In a previous randomised controlled trial, Wang et al. [6] demonstrated that LCDs can improve blood glucose more than a low-fat diet in Chinese patients with type 2 diabetes. In a separate randomised controlled study, the effects of almond-based LCD on depression, glycometabolism, gut microbiota, and glucagon-like peptide-1 in Chinese patients with type 2 diabetes were examined [5]. In this study, it was found that LCD could exert a beneficial effect on depression and glycometabolism in Chinese patients with type 2 diabetes [5]. However, it remains unclear whether these findings can be replicated in other populations.

Furthermore, the definition of LCDs and their use in managing people with diabetes have been seen as controversial [4,9]. For example, different definitions of LCDs have been reported in the literature. LCDs have sometimes been defined as consisting of <30% carbohydrates of total energy intake or 100 g/day carbohydrate, while others have defined LCDs as providing 50–150 g/day carbohydrates [9]. Despite the broadly agreed definition of LCDs as <26% carbohydrates of total energy intake or <130 g/day carbohydrate, this definition is not being implemented consistently in research globally [9]. Therefore, it is difficult to compare the findings and make recommendations to support people with type 2 diabetes [9].

The controversy in the use of LCDs may be due to the fact that the most effective quantity of carbohydrate intake for people with type 2 diabetes has not been determined, and the higher fat and protein content in LCDs has raised concerns in terms of safety [10]. For example, there are no recommendations on carbohydrate intake specifically for people with type 2 diabetes in the UK, and thus, they rely on the advice for the general UK population [8]. However, reducing the total carbohydrate intake as a strategy for improving glycaemic control in people with diabetes has been established [10]. There have also been conflicting findings about the lasting effects of LCD on metabolic parameters in people with type 2 diabetes [10]. In this regard, while Li et al. [10] results demonstrated a more lasting effect of LCDs on glycaemic control, other studies [11,12] have observed rebounds of glycated haemoglobin after 6 or 12 months of intervention. Therefore, there is a need to examine current trends in the use of low-carbohydrate diet in managing people with type 2 diabetes.

Aim: To examine current advances and future prospects in the use of LCD in managing people with type 2 diabetes

Research Question: What are the current trends and future perspectives in the use of LCD in managing people with type 2 diabetes?

## 2. Method

The Preferred Reporting Items for Systematic Reviews and Meta-Analyses (PRISMA–Supplementary Table S1) was applied to report the systematic review [13]. The protocol for the systematic review was registered with PROSPERO (Registration Number: CRD420251040421).

### 2.1. Population: Adults Diagnosed with Type 2 Diabetes

Outcomes of Interest: Outcomes included glycated haemoglobin, fasting blood glucose, postprandial blood glucose, lipid profile, and Body Mass Index (BMI).

### 2.2. Search Strategy

The following databases, including MEDLINE, APA PsycInfo, Academic Search Premier, CINAHL Plus with Full Text, APA PsycArticles, and Psychology and Behavioural Sciences Collection, were searched via EBSCOHost. The EMBASE database and reference list of articles were also searched for articles of interest. The authors relied on the Population, Intervention, Comparator, Outcomes, and Study (PICOS) framework to address the search terms and the research question (Table 1). Two researchers (OO and OOO) conducted the searches independently, from the database inception to 28 August 2025. However, based on the inclusion criteria, the year of publication of studies included was restricted to articles published from 2021 to date. The terms used for searches conducted in the EBSCOHost database and EMBASE database are outlined in Table 1, under the headings of Population, Intervention, and Study Design. The search terms in each column (Table 1) were combined using the Boolean operator (OR). The results of the searches in each column were then combined using the Boolean operator (AND), in searches conducted separately in the EBSCOHost database and EMBASE database. The duplicates were removed in EndNote (Analytics, Philadelphia, PA, USA).

### 2.3. Data Collection

**Figure 1:**
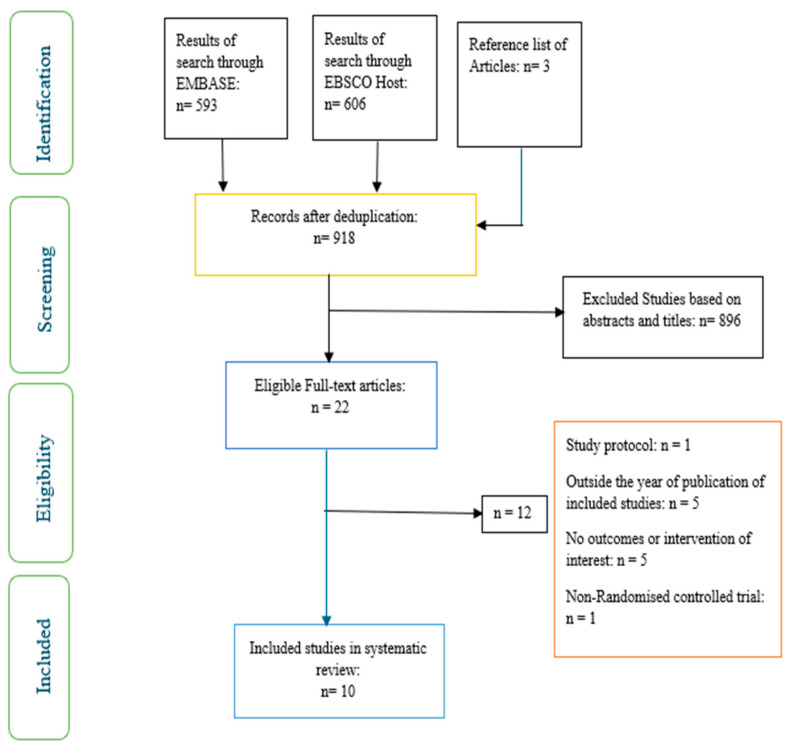
PRISMA flow chart of studies included.

The articles were screened for eligibility based on inclusion and exclusion criteria by two researchers (OO and OOO) (Figure 1). The researchers screened the articles independently and resolved differences through discussion.

### 2.4. Study Selection

Inclusion Criteria: Only randomised controlled studies, studies involving patients with type 2 diabetes, and written in English were included in the review. Only articles published from 2021 to the date of search were included in order to gain current trends in the use of LCDs in people with type 2 diabetes.

Exclusion criteria: Adolescents (under 18 years of age) with type 2 diabetes, studies with LCDs combined with other interventions such as exercise or high protein, involving patients with type 1 or gestational diabetes, or publication of protocols or only an abstract were excluded from the review.

### 2.5. Data Extraction and Management

The extraction of data from the included studies was independently completed by two researchers (YO and VA). Differences between the researchers were resolved through discussion. The following characteristics of the included studies, such as research method, citation, the aim of the study, the mean age, the sample size, and the results, were extracted from the studies (Table 2). The findings were synthesised using narrative synthesis [14].

### 2.6. Assessment of Risk of Bias

The Risk of Bias (Quality) of included studies was assessed using the domain-based risk evaluation tool [15]. The domains that were assessed included attrition bias, allocation concealment (selection bias), reporting bias, detection bias, selection bias, and performance bias.

### 2.7. Outcomes

The main outcomes of interest were glycated haemoglobin, fasting blood glucose, postprandial blood glucose, lipid profile, and body mass index.

**Table 2:** Description of the included studies.

Study/Country of Study	Study Design	Sample Size	Age (Years)	Aim/Objective	Interventions	Results	Conclusion
Al-Ozairi et al. [16] Kuwait	Randomised crossover trial	n = 15 started the study Male: n = 9 Female: n = 6 n = 12 (completed)	47–56 years Mean: 54 years All diagnosed with T2D within 4 years	To explore the dose–response effect of carbohydrate restriction (10–30% kcal) on glycaemia in people with well-controlled type 2 diabetes, while keeping calories and protein constant and preventing weight loss.	Intervention: Five different 6-day eucaloric diets with varying carbohydrate content: 10%, 15%, 20%, 25%, 30% of total kcal (protein kept constant at 15% kcal, remainder fat). All food provided; daily self-weighing to ensure weight stability; 7+ day washout between arms. Glycaemia measured by continuous glucose monitoring (CGM).	Primary outcome: No significant differences in 24-h mean glucose (7.4 ± 1.1 mmol/L at 10% versus 7.6 ± 1.3 mmol/L at 30%, *p* = 0.28) or postprandial glucose at 10% (8.1 ± 1.5 versus 8.5 ± 1.4 mmol/L, at 30% *p* = 0.28) between the highest and lowest carb doses. No dose–response relationship observed. Small weight loss occurred in all arms (0.4–1.1 kg over 6 days), but adjusting for this did not change results.	Reasonable changes in dietary carbohydrate content (10–30% kcal) do not influence glycaemic control in people with well-controlled T2D when weight and protein intake are kept constant. Carbohydrate restriction alone, without weight loss or increased protein, may not lower glucose in people with well-controlled diabetes over the short-term period.
Alzahrani et al. [17] Denmark	Extension of a prior randomised crossover 6-month open-label prospective follow-up	28 with T2D Male (n = 20) Female (n = 8)	64 ± 7.7	To assess whether the beneficial effects of a carbohydrate-reduced, high-protein (CRHP) diet on cardiovascular risk markers in T2D are maintained when patients prepare their own food, with dietitian support, over 6 months	CRHP diet: 30% carbs, 30% protein, 40% fat (self-prepared, weight-maintaining, dietitian-supported diet). Control: 50% carbs, 17% protein 33% fat (consistent with European dietary guidelines)	At 6 months (week 36) compared with baseline: Significant reduction in: fasting total cholesterol (*p* < 0.05) LDL cholesterol (*p* < 0.05) Fasting & postprandial NEFA and TG: (*p* < 0.05) Fasting apoB, CRP, TNF-α: (*p* < 0.05) Changes were independent of minor body weight fluctuations.	Substituting dietary carbohydrate for protein and fat, in a real-life setting under dietitian guidance, has beneficial effects on multiple cardiovascular risk markers in patients with T2D, which are maintained or improved over 6 months, when patients prepare their own CRHP diet.
Chen et al. [18] Taiwan	1-year follow-up RCT after 18-month open-label RCT	71 LCD (n = 36) TDD Traditional diabetic diet (n = 35)	LCD 63.3 ± 10.9 63.2 ± 6.8	To evaluate the effect at 1-year follow-up after an 18-month RCT of a 90 g/day LCD in poorly controlled type 2 diabetes patients.	LCD: <90 g/day carbs, no energy restriction. TDD: 50–60% carbs, 1.0–1.2 g/kg protein, ≤30% fat, (Daily calorie intake was tailored to individual BMI). Both groups had regular follow-up.	At 30 months, LCD group consumed less carbohydrate than TDD group. (131.8 ± 53.9 g versus 195.1 ± 50.2 g, *p* < 0.001) LCD has lower HbA1c (7.2% versus 7.7%, *p* = 0.017), lower 2-h postprandial glucose (*p* < 0.001), lower ALT (*p* = 0.017), No significant differences in % change of fasting glucose, total cholesterol, triglycerides, LDL, HDL, BMI, or weight between groups from 18 to 30 months (*p* > 0.05).	A 90 g/day LCD showed a better glycaemic control, liver function, and lower medication need than TDD at 30 months in poorly controlled T2D. However, the improvement in glycaemia and lipid profile between 18 and 30 months was similar between groups, suggesting that a sustained lower carb intake can be beneficial to improve glycaemic control in poorly controlled T2D.
Dening et al. [19] Australia	Parallel RCT	Randomised (n = 98) Intervention Web-based T2Diet programme plus standard care (n = 49) withdrawal (n = 9) Control Standard care only (n = 49) withdrawal (n = 2) Analysis n = 87 Intervention n = 40 Control n = 47	Intervention group 61.3 ± 9.4 Control 59.8 ± 9.6	To evaluate whether a web-based LCD programme provided in conjunction with standard care improves glycaemic control in adults with T2D.	Intervention: Web-based LCD education (50–100 g CHO/day), high intake of non-starchy vegetables, dietary fibre + standard care Control: Standard care only	At 16 weeks, a significant reduction in the intervention group compared with the control group was reported in Glycaemic Parameters: HbA1c: −0.65% (95% CI: −0.99 to −0.30; *p* < 0.0001) Fasting & postprandial glucose: Not specifically reported Significant reduction in the intervention group was reported in BMI and weight. BMI: −1.11 kg/m^2^ (*p* < 0.0001) Weight: −3.26 kg (*p* < 0.0001).	In addition to standard care in adults with T2D, the web-based LCD intervention significantly improved glycaemic control (HbA1c), BMI, and weight compared to standard care only. The web-based dietary education and support programme highlights the potential of improving accessibility available for people with T2D to achieve glycemic control and improve diabetes outcomes.
Dorans et al. [20] USA	Randomized Clinical Trial	150 Low-carb diet (n = 75) Usual diet (n = 75)	40–70 years with untreated HbA1c of 6.0% to 6.9% (42–52 mmol/mol) Low-carb diet 59.3 ± 7.0 Usual diet 58.6 ± 8.8	To assess the effect of a behavioral intervention promoting a low-carbohydrate diet compared with a usual diet on 6-month changes in HbA1c among adults with elevated untreated HbA1c (6.0–6.9%).	Low-carb diet group: Target <40 g net carbs/day (first 3 months), <60 g net carbs/day (months 4–6), with counseling. Usual diet group: Standard dietary advice, no ongoing recommendations.	At 6 months, in the low-carb group compared with the usual diet group, HbA1c: Net 6-month reduction was −0.23% (95% CI, −0.32% to −0.14%; *p* < 0.001) Fasting plasma glucose: Net reduction −10.3 mg/dL (95% CI, −15.6 to −4.9; *p* < 0.001). Body weight: Net reduction −5.9 kg (95% CI, −7.4 to −4.4; *p* < 0.001). BMI: Net reduction −2.0 (95% CI, −2.5 to −1.5; *p* < 0.001). Lipid profile: No significant differences between groups in total cholesterol, LDL, HDL, or triglycerides (all *p* > 0.05).	Compared to a usual diet, a low-carbohydrate dietary intervention significantly contributes to a reduction in glycaemic parameters (HbA1c, fasting glucose), and BMI and body weight among adults with elevated untreated HbA1c (6.0–6.9%), but effects independent of weight loss could not be determined. No significant effect on lipid profile. If a low-carb diet is sustained, it may help prevent and treat type 2 diabetes.
Gram-Kampmann et al. [21] Denmark	Open-label RCT	71 Intervention: LCD n = 49 Control: n = 22	Data: mean ± SEM Intervention: 57.3 ± 0.9 Control: 55.2 ± 2.7	To investigate the efficacy and safety of a non-calorie–restricted LCD on glycaemic control, body composition, and cardiovascular risk factors in patients with type 2 diabetes, while maintaining their non-insulin antidiabetic medication and physical activity.	Intervention: LCD ≤20% energy from carbs 50–60% fat 25–30% protein. Control: (official Danish dietary guidelines) 50–60% carbs, 20–30% fat, 20–25% protein. Both groups were non-calorie–restricted and maintained physical activity and medication.	At 6 months, Primary outcome: Compared with the control group, there is a reported significant reduction in HbA1c with LCD HbA1c: −7.5 ± 1.8 mmol/mol (*p* < 0.0001) Secondary outcomes: Significant reductions in BMI: −1.4 ± 0.4 kg/m^2^; Weight: −3.9 ± 1.0 kg; and waist circumference: −4.9 ± 1.3 cm (all *p* < 0.001). No significant changes in blood lipids or blood pressure. No episode of severe hypoglycaemia	A non-calorie–restricted LCD high in fat significantly improves glycaemic control and body composition without adversely affecting cardiovascular risk factors or causing severe hypoglycaemia in T2D patients. Reducing carbohydrate intake to 10–25% of energy is an effective and safe nutritional approach for this population.
Gram- Kampmann et al. [22] Denmark	Open-label RCT	70 LCD (n = 49) Control (n = 21) Ratio 2:1 64 completed at 6 months	LCD 55.2 ± 6.2 Control 57.1 ± 12.9	To assess whether a non-calorie-restricted LCD high in fat adversely affects endothelial function (FMD/NID) and markers of low-grade inflammation (hsCRP, IL-6) in patients with type 2 diabetes.	LCD: <20% energy from carbs, 50–60% fat, 25–30% protein, with emphasis on high intake of MUFA and low SFA. Control: 50–60% carbs, 20–30% fat, 20–25% protein, <10% SFA (current official Danish dietary guidelines) Both groups received dietitian support and were advised to maintain their weight and physical activity.	Primary outcomes at 6 months, FMD and NID: No significant changes in both groups after 6 months; no between-group differences (FMD *p* = 0.34, NID *p* = 0.53). Inflammatory markers: hsCRP and IL-6 decreased significantly only in the LCD group (*p* < 0.05), but between-group differences were not statistically significant (hsCRP *p* = 0.07, IL-6 *p* = 0.25). There is no change in results after adjustment for risk factors. No change in results.	A 6-month LCD high-fat diet does not adversely affect endothelial function or selected markers of low-grade inflammation in T2D, suggesting this nutritional approach does not increase cardiovascular disease risk in type 2 diabetes patients.
Hansen et al. [23] Denmark	RCT	165 participants with T2D LCD (n = 110) HCLF (n = 55) Ratio 2:1	LCD 57 ± 9 HCLF 55 ± 12	To investigate the effect of a calorie-unrestricted low-carbohydrate, high-fat (LCD) diet on type 2 diabetes mellitus (T2D) and nonalcoholic fatty liver disease (NAFLD), compared with a high-carbohydrate, low-fat (HCLF) diet	Two calorie-unrestricted diets: LCD: ≤20% carbs 25–30% protein 50–60% energy from fat. HCLF: 50–60% carbs, 20–25% protein 20–30% fat	At 6 months, LCD group HbA1c improved more in the LCD group than in the HCLF group (mean difference in change: −6.1 mmol/mol (95% CI, −9.2 to −3.0 mmol/mol) or −0.59% (95% CI, −0.87% to −0.30%) equivalent. Fasting blood glucose showed improvement in LCD LCD has improved HDL and triglycerides but raised LDL cholesterol (mean difference: 0.37 mmol/L or 14.3 mg/dL) compared to HCLF No significant between-group changes in NAFLD assessment. BMI: LCD led to greater mean weight loss −3.8 kg (95% CI, −6.2 to −1.4 kg) compared with HCLF group.	A calorie-unrestricted LCD diet led to improvements in glycaemic control (HbA1c and fasting glucose) and weight in T2DM compared to an HCLF diet, but these changes were not sustained 3 months after intervention.
McCullough et al. [24] UK	Parallel Randomised Design	n = 16 participants LCD (n = 8) Male n = 4 Female n = 4 HCLF (n = 8) Male n = 5 Female n = 3	LCLF 43.8 ± 10.4 HCLF 44.6 ± 15.27	To investigate the impact of an ad libitum 8-week low-carbohydrate, high-fat (LCD) diet compared with a high-carbohydrate, low-fat (HCLF) diet on cardiometabolic risk factors, the plasma metabolome, and markers of glucose and insulin metabolism in adults with a slightly elevated cardiometabolic risk.	8 weeks duration: LCD: ≤50 g carbohydrate/day, increased fat, protein same as HCLF HCLF: 50% carbohydrate, 15% protein, ≤35% fat (UK Eatwell Guide), high fibre, low free sugars Both diets ad libitum (no calorie restriction)	After 8 weeks, Glycaemic changes: both an LCD and an HCLF diet significantly (*p* < 0.01) improved fasting insulin, HOMA IR, rQUICKI, and leptin/adiponectin ratio (*p* < 0.05) levels. LCD group showed upregulation in lipid metabolites, indicating increased lipid transport and oxidation. −78 metabolites were differentially regulated between groups. Both diets may reduce T2D risk.	The markers of insulin resistance and metabolic risk can improve with both LCD and HCLF diets. However, as indicated by metabolomic profiling, an LCD diet may further enhance insulin sensitivity by promoting lipid oxidation.
Oliveira et al. [25] Canada and Australia	2 sites- Parallel RCT	121 Intervention: Low-carb breakfast (LC) n = 60 Control: Low-fat control breakfast (CTL) n = 61	Intervention: 65 ± 9 Control: 64 ± 10	To determine if having a low-carbohydrate (LC) breakfast compared to a low-fat (CTL) breakfast improves glycemic control in people with type 2 diabetes (T2D) over 3 months.	Intervention: LC breakfast: ~465 kcal (25 g protein, 8 g carbs, 37 g fat), for example, omelet with cheese and non-starchy vegetables Control breakfast: ~450 kcal (20 g protein, 56 g carbs, 15 g fat)—for example, oatmeal and fruit-based. No specific dietary guidance or calorie restriction for other meals.	At 12 weeks, primary outcome: HbA1c reduced by −0.3% (95% CI: −0.4%, −0.1%) in the LC group; between-group difference was borderline statistically significant (−0.2%, (95% CI: −0.4%, 0.0%), *p* = 0.06). LC breakfast led to a reasonable but clinically relevant reduction in HbA1c. Fasting Blood Glucose: No significant difference in fasting glucose between the LC and control groups at 12 weeks. Postprandial Blood Glucose: Compared to the control group, the LC group had significantly lower post-breakfast 2-h glucose, mean and maximum glucose, and glycaemic variability (all *p* < 0.05). The LC group also had lower daily energy and carbohydrate intake. Lipid Profile: No significant differences reported BMI: Both groups had a small reduction in self-reported body weight, (−0.1 (−1.6 to 1.5) but no significant difference between groups (*p* = 0.92).	LC breakfast is a simple, realistic strategy to reduce energy and carbohydrate intake and improve several continuous glucose monitoring variables in people living with T2D, without adverse effects on cholesterol or weight compared to a low-fat control breakfast.

Abbreviations: alanine aminotransferase (ALT); apolipoprotein B (apoB); body mass index (BMI); carbohydrate (Carbs, CHO); carbohydrate-reduced, high-protein (CRHP); cardiovascular (CVS); confidence interval (CI); continuous glucose monitoring (CGM); control (CTL); C-reactive protein (CRP); flow-mediated vasodilation (FMD); heamoglobin A1c (HbA1c); high-carbohydrate, low-fat (HCLF); high-density lipoprotein (HDL) cholesterol; high sensitivity CRP (hsCRP); homeostatic model of insulin resistance (HOMA IR); interleukin-6 (IL-6); low-carbohydrate diet (LCD); low-carbohydrate, high-fat (LCD); low-density lipoprotein (LDL) cholesterol; means ± standard deviation (SD); monounsaturated fatty acids (MUFAs); nitroglycerine induced dilation (NID); nonalcoholic fatty liver disease (NAFLD); non-esterified fatty acid (NEFA); randomised controlled trial (RCT); revised Quantitative Insulin sensitivity Check Index (rQUICKI); saturated fatty acids (SFA); traditional diabetic diet (TDD); triacylglycerol (TG); tumour necrosis factor-alpha (TNF-α); type 2 diabetes (T2D).

### 2.8. Risk of Bias Assessment of Included Studies

**Figure 2:**
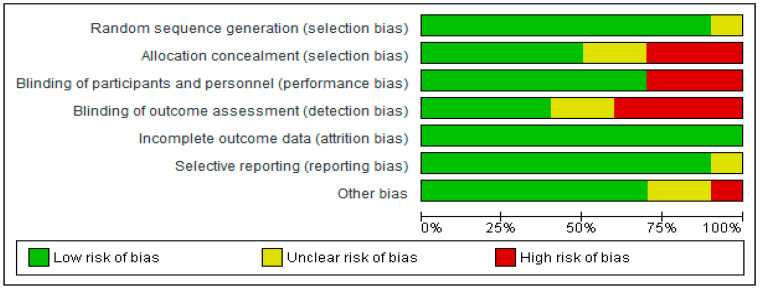
Risk of bias graph of included Studies [16,17,18,19,20,21,22,23,24,25].

**Figure 3:**
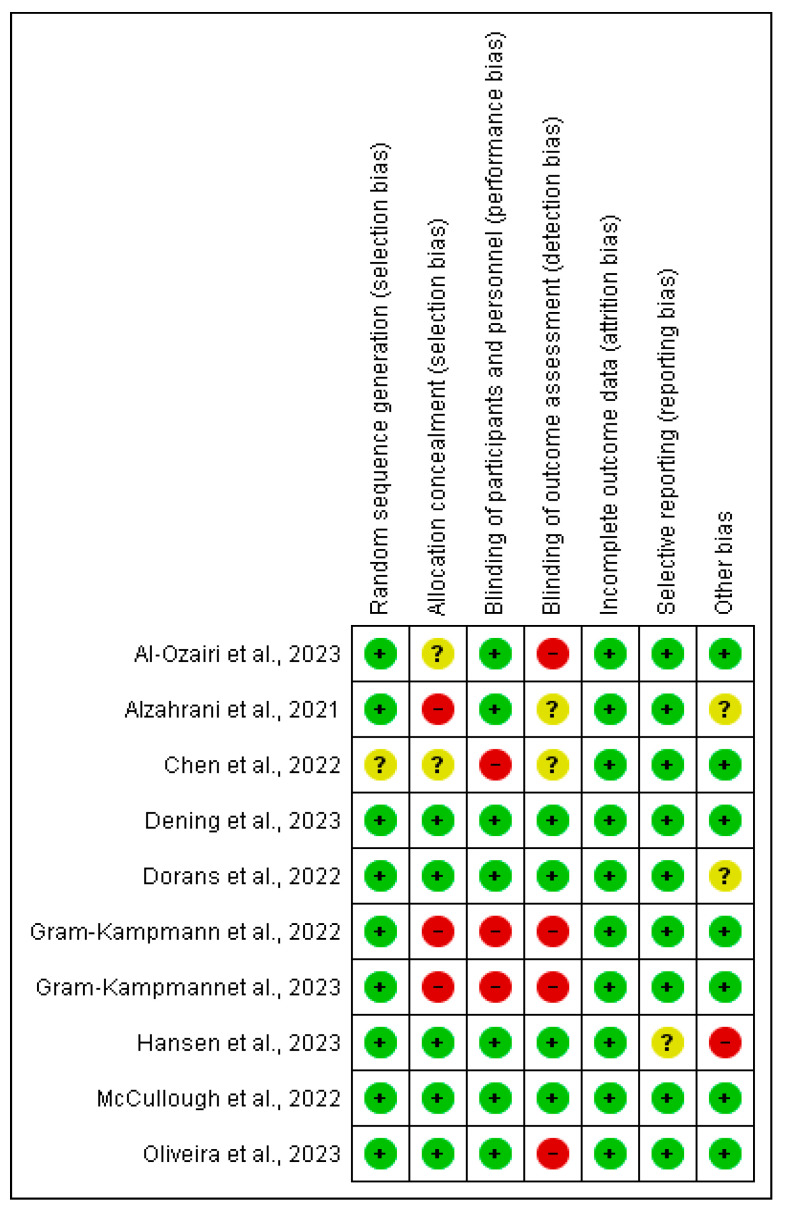
Risk of bias summary of included Studies [16,17,18,19,20,21,22,23,24,25].

With respect to incomplete outcome data, there was a low risk of bias in all the studies included (Figure 2 and Figure 3). Random sequence generation and selective reporting had low risk of bias in the majority of studies except Chen et al. [18] and Hansen et al. [23], where there was an unclear risk of bias in relation to these two domains, respectively (Figure 2 and Figure 3).

In relation to allocation concealment, five studies had a low risk of bias, two studies [16,18] had an unclear risk of bias, while three studies [17,21,22] had a high risk of bias. Blinding of participants and personnel was of a low risk of bias in seven of the studies, while the remaining three studies [18,21,22] were assessed as having a high risk of bias. With respect to blinding of outcome assessment, while four of the studies had a low risk of bias, another four studies [16,21,22,25] had a high risk of bias, and the remaining two studies [17,18] had an unclear risk of bias.

Seven studies had a low risk of bias in the ‘Other bias’ domain; two additional studies [17,20] were assessed as having an unclear risk of bias, while one study [23] had a high risk of bias in this domain (Figure 2 and Figure 3).

### 2.9. Findings

The narrative synthesis of findings evaluates the impact of LCD interventions on glycated haemoglobin (HbA1c), fasting blood glucose, postprandial blood glucose, lipids, and BMI across ten RCTs conducted in diverse populations with type 2 diabetes. The studies varied in design, duration (ranging from 6 days to 30 months), and dietary composition, yet collectively provided valuable insights on all measured outcomes.

### 2.10. Glycated Haemoglobin (HbA1c)

Of the ten studies, four did not report HbA1c as an outcome [16,17,22,24], and the findings of one study were reported across two papers [21,22], of which only one presented HbA1c [21]. The impact of LCD interventions significantly impacted HbA1c levels across different durations, ranging from three to six months; however, the impact did not appear sustainable over longer time periods.

At three months, HbA1c was significantly reduced in those following a LCD −0.3% (95% CI: −0.4%, −0.1%), although there was no significant difference between the LCD and control group −0.2; (95% CI: −0.4, 0.0; *p* = 0.06) [25]. At 16 weeks, HbA1c was significantly reduced in those following a LCD and a significant difference was identified between the LCD and control group −0.65% (95% CI: −0.99 to −0.30; *p* < 0.0001) [19]. The significant reduction of HbA1c at three months in the LCD group (β = −8.9 ± 1.7 mmol/mol; *p* ≤ 0.0001) was sustained at six months (β = −7.5 ± 1.7 mmol/mol; *p* ≤ 0.0001) compared to the control group [21]. A further analysis, which excluded those prescribed insulin, sulphonylureas, and glucose-lowering drugs, did not reduce the effect of the LCD at three and six months (*p* < 0.0001) [21].

At six months HbA1c was significantly reduced between the LCD and control, with a net reduction of −0.23% (95% CI, −0.32% to −0.14%; *p* < 0.001) and the LCD group had a larger reduction in HbA1c than the control with a net difference in change of −0.23% (95% CI, −0.32% to −0.14%; *p* < 0.001) [20]. Also, at six months, HbA1c was significantly reduced for the LCD group compared to the HCLF group with a mean difference in change of −6.1 mmol/mol (95% CI, −9.2 to −3.0 mmol/mol) [23]. However, at nine months, the HbA1c had returned to baseline levels for both groups [23]. Lastly, HbA1c at 18 and 30 months for both the LCD and TDD remained significantly lower (*p* < 0.05) than baseline; however, HbA1c had significantly increased in the LCD group (*p* < 0.05) between 18 and 30 months, and there was no significant difference between groups at both 18 and 30 months [18].

### 2.11. Fasting Blood Glucose

Of the ten studies, two did not report fasting blood glucose levels as an outcome [17,19], and the findings of one study were reported across two papers [21,22], of which only one presented fasting glucose levels [21]. The impact of different dietary interventions on fasting blood glucose levels across the eight remaining studies varied considerably; two studies identified no effect of LCD on fasting glucose levels [21,23]. One study identified no significant difference between fasting glucose levels between the intervention and control group [25], and two studies identified no significant difference between the different interventions [16,18]. Two studies identified a significant decrease in fasting glucose levels at 6 months, firstly, Dorans et al. [20] identified a net reduction −10.3 mg/dL (95% CI, −15.6 to −4.9; *p* < 0.001) in the low-carbohydrate intervention group, secondly Hansen et al. [23] identified a reduction mg/dL −25.41 (95% CI, −30.09 to −20.72) in the LCD group and −10.63 (95% CI, −16.94 to −4.32) in the HCHF group (*p* < 0.001), however, this was not sustained at 9 months.

### 2.12. Postprandial Blood Glucose

Of the ten studies, five did not report postprandial blood glucose levels as an outcome [17,19,20,23,24], and the findings of one study were reported across two papers [21,22], of which only one presented postprandial blood glucose [21]. The impact of dietary interventions on postprandial blood glucose varied considerably. Following an LCD, postprandial blood glucose was significantly lower two hours post breakfast when compared to a control group (−2.2 mmol/L; 95% CI: −3.0, −1.4 mmol/L; *p* < 0.01) [25]. Self-reported postprandial blood glucose at three and six months demonstrated a reduction for those in the LCD group compared to the control, except for reading post-lunch [21]. Postprandial blood glucose was also significantly lower for both the LCD and TDD groups and remained significantly lower (*p* < 0.05) at 18 and 30 months; however, postprandial blood glucose had significantly increased in the LCD group (*p* < 0.05), and no significant difference between groups remained at both 18 and 30 months [18]. One study identified no differences between dietary interventions and postprandial blood glucose [16]. For example, the results of the 30% kcal and 10% kcal doses were 8.1 ± 1.5 mmol L^−1^ vs. 8.5 ± 1.4 mmol L^−1^ (*p* = 0.28), respectively, and there was no dose–response relationship between the dose of carbohydrates and postprandial blood glucose (*p* > 0.05) [16].

### 2.13. Lipids Profile

Of the ten studies, two did not report lipid profiles, such as cholesterol, HDL, LDL, and triglycerides [16,19]. Three studies identified improvement in lipid profile. Hansen et al. [23] reported that after six months, LCD had improved HDL and triglycerides but raised LDL cholesterol with a mean difference of 0.37 mmol/L or 14.3 mg/L compared to HCLF. Similarly, McCullough et al. [24] reported that the LCD group showed upregulation in lipid metabolites, indicating increased lipid transport and oxidation; and 78 metabolites were differently regulated between groups after 8 weeks. Alzahrani et al. [17] at six months also reported a statistically significant increase in HDL (*p* < 0.05) and apoA1 concentrations, and a reduction in LDL (*p* < 0.05).

However, five studies reported no significant changes, such as Gram-Kampmann et al. [21], who reported no significant changes in blood lipids, whereas Gram-Kampmann et al. [22] found that inflammatory markers-hsCRP and IL-6 decreased significantly only in the LCD group (*p* < 0.05) but were not statistically significant between groups after six months. Doran et al. [20] compared the low-carb group with the usual diet group and reported no significant differences at six months between groups in total cholesterol, LDL, HDL, or triglycerides (>0.05). Similarly, Chen et al. [18] and Oliveira et al. [25] reported no significant change in fasting blood, total cholesterol, triglycerides, LDL, and HDL.

### 2.14. Body Mass Index

Among the ten studies, five reported statistically significant reductions in BMI or body weight in the LCD groups compared to controls. Dorans et al. [20] observed a BMI reduction of 2.0 kg/m^2^ over six months, while Dening et al. [19] reported a 1.11 kg/m^2^ decrease following a 16-week web-based LCD intervention. Gram-Kampmann et al. [21] found a 1.4 kg/m^2^ reduction over six months with a non-calorie-restricted LCD, and Hansen et al. [23] reported the most substantial weight loss (−3.8 kg) in the LCD group over six months. These findings suggest that LCD can effectively reduce BMI, even in the absence of explicit calorie restriction, likely due to spontaneous reductions in energy intake and improved satiety.

Conversely, several studies reported no significant changes in BMI. Oliveira et al. [25], which focused on a low-carb breakfast intervention, and Chen et al. [18], a long-term follow-up study, both found no significant BMI differences between LCD and control groups. Similarly, Alzahrani et al. [17] and Gram-Kampmann et al. [22] observed stable BMI values, despite improvements in cardiovascular and inflammatory markers. Al-Ozairi et al. [16], using a tightly controlled crossover design, also reported no BMI change, reinforcing the importance of energy balance in weight outcomes.

### 2.15. Emerging Themes

1. Duration and dietary scope are critical

Short-term LCD interventions ranging from three to six months significantly impacted HbA1c levels [20,23,25]. However, the impact did not appear sustainable over longer time periods [18]. Short-term (3 months) and long-term (18–30 months) LCD interventions did not significantly impact fasting blood glucose levels [18,25]. However, the impact was significant at six months [20,23]. Short-term and long-term LCD interventions significantly reduced postprandial blood glucose [21,25]. However, the impact of long-term LCD intervention compared to a control demonstrated no significant difference between groups [18]. Short-term and long-term LCD interventions did not significantly impact lipid profile levels [18,20,21]. However, at six months, a significant difference in lipid profile levels was identified [17,23,24]. Short-term or single-meal interventions [16,25] did not significantly affect BMI, highlighting the importance of sustained, whole-diet approaches for achieving weight loss. However, longer interventions [20,23] were more likely to yield significant BMI reductions.

2. Feasibility and sustainability of LCDs

The feasibility of LCDs was identified through the approach of web-based programmes, which improved access and availability of care to people with type 2 diabetes [19]. Furthermore, self-prepared LCDs, supported by dietitians, were both feasible and effective in real-world settings, offering scalable solutions for management of type 2 diabetes [17,19]. The feasibility and sustainability of LCDs have been suggested through the approach of simple dietary advice to reduce carbohydrate intake by people with type 2 diabetes [25]. Alongside a safe nutritional approach to sustainable outcomes of prolonged and better glycaemic control for people with type 2 diabetes [18], achieve glycaemic control and improved health [19], improve insulin sensitivity [24], and a useful dietary approach for both preventing and treating type 2 diabetes [20].

## 3. Discussion

The findings of this systematic review have shown that a low-carbohydrate diet is significantly (*p* < 0.05) more effective in reducing glycaemic parameters compared to a usual diet, standard care, or control diet in people with type 2 diabetes [18,19,20,21,23,24]. Similarly, the effect of a low-carbohydrate diet was significant (*p* < 0.05) in reducing BMI in these patients compared with a control diet [19,20,22]. However, a low-carbohydrate diet did not appear to have a significant (*p* > 0.05) effect on lipid parameters compared to a control diet [18,19,20,21,25].

The results of this review confirm the findings of an earlier systematic review by Siregar et al. [26] and a comprehensive review by Pavlidou et al. [27] with respect to glycaemic control. For example, Siregar et al. [26] reported that a low-carbohydrate diet was effective in reducing glycated haemoglobin, controlling blood glucose, and improving quality of life in patients with type 2 diabetes. Similarly, Pavlidou et al. [27] noted there was significant evidence to support the effectiveness of a low-carbohydrate diet in people with diabetes. Various randomised controlled trials [5,6,28] and an experimental study [29] have also found that low-carbohydrate diets were significantly more effective compared with control diets in reducing blood glucose parameters and BMI in patients with type 2 diabetes. Li et al. [10] observed that low-carbohydrate diets were more effective compared with low-fat diets in improving glycated haemoglobin and reducing body weight in people with type 2 diabetes.

In our current review, we found that a low-carbohydrate diet was also significantly more effective than a control diet in reducing BMI, which reaffirms the findings of the review by Pavlidou et al. [27], which showed that a low-carbohydrate diet can reduce BMI and total body fat mass. In a subgroup analysis conducted by Lei et al. [30], no significant difference was found between a low-carbohydrate diet and a low-fat diet with respect to lipid parameters, including total cholesterol and low-density lipoprotein cholesterol in participants with diabetes. In addition, Chen et al. [31], examined the effect of a 90 g/day low-carbohydrate diet in people with type 2 diabetes in an 18-month randomised controlled study, and found the low-carbohydrate diet provided better effect (*p* < 0.05) compared with control diet, with respect to glycaemic control, although differences between the two groups were not significant (*p* > 0.05) with respect to lipid profile. These earlier findings by Lei et al. [30] and Chen et al. [31] have been confirmed based on the results of the current systematic review.

The possible mechanisms of action of a low-carbohydrate diet in the control of blood glucose parameters and BMI have been reported [11]. It has been suggested that the greater reduction in glycated haemoglobin in people on a low-carbohydrate diet may be due to increased weight loss [11]. The increased weight loss in people on a low-carbohydrate diet may also explain the significant difference observed between the low-carbohydrate diet and control groups with respect to BMI. Ren et al. [5] also found that an almond-based low-carbohydrate diet significantly decreased body weight and BMI in people with type 2 diabetes. The decrease in weight and BMI in people with type 2 diabetes on a low-carbohydrate diet may increase insulin sensitivity in this group and thus lead to improved glycaemic control [32].

One of the possible effects of carbohydrate reduction is the oxidation of fat for energy, which could lead to the loss of body fat stores and the production of ketone bodies that induce satiety [11]. According to Currenti et al. [29], the reduction in adipose tissue in people with type 2 diabetes on low-carbohydrate diets decreases the release of pro-inflammatory adipokines, including tumour necrosis factor alpha and interleukin 6, which have been reported to contribute to low-grade systemic inflammation and insulin resistance.

Carbohydrates, being the primary macronutrient that influences postprandial blood glucose, their reduction in low-carbohydrate diets leads to reduced glucose availability, resulting in lower postprandial glucose excursions and overall glucose levels [11,29]. Therefore, a low-carbohydrate diet may promote reduced demand for insulin secretion, thus leading to improved glucose control [29].

### Limitations of the Review

The inclusion criteria, which restricted the articles included in this review to only studies published from 2021, may have limited the number of studies in this review to only ten and the broader application of its findings.

Furthermore, some of the studies included in this review [16,17,18,21,22,23,25] had high or unclear risk of bias in some of the risk of bias domains, such as allocation concealment, blinding of participants and personnel, and blinding of outcome assessment. These limitations have implications as they could potentially lead to exaggerated estimates of the effects of the treatments, thus distorting the results of the studies.

Some of the studies included in this review have potential limitations, such as, the studies involving five 6 day isocaloric diets with varying proportions of carbohydrates [16], 6 months follow up of previous RCT where participants were provided with pre-packaged ready to eat food during the initial 12 weeks [17], 1 year follow-up after 18 months RCT [18] and the study with only low-carbohydrate breakfast [25].

## 4. Conclusions

This systematic review found that a low-carbohydrate diet is significantly (*p* < 0.05) more effective in promoting glycaemic control than a usual diet, standard care, or control diet in people with type 2 diabetes. In addition, LCDs can be an effective strategy for reducing BMI in individuals with type 2 diabetes, particularly when implemented as part of a structured, sustained dietary intervention. However, there was variability in the findings of the studies included with respect to glycaemic control and BMI, and the impact of LCDs on glycaemic control did not appear sustainable in the long term. The impact of LCDs on BMI appears to be influenced by factors such as intervention duration, energy intake, adherence, and dietary composition. These findings support the integration of LCDs into personalised nutrition strategies for type 2 diabetes management, with attention to long-term sustainability and individual metabolic responses. However, a low-carbohydrate diet did not appear to have a significant (*p* > 0.05) effect on lipid parameters compared to the control diet.

## Figures and Tables

**Table 1:** Search Strategy.

Population	Intervention	Study Design	Search Terms Combined
Patients with diabetes OR type 2 diabetes OR diabetes OR diabetes complications OR diabetes mellitus, type 2 OR diabetes mellitus	low-carbohydrate diet or low-carbohydrate or low-carb diet	Randomised controlled trial OR controlled clinical trial OR randomized OR placebo OR drug therapy OR randomly OR trial OR groups	Columns 1, 2, and 3

## Data Availability

Not applicable.

## Supplementary Materials

The following supporting information can be downloaded at: https://www.mdpi.com/article/10.3390/ijerph22091352/s1, Table S1: Checklist.

## Abbreviations

ALT	alanine aminotransferase
apoB	apolipoprotein B
BMI	body mass index
Carbs, CHO	carbohydrate
CRHP	carbohydrate-reduced, high-protein
CVS	cardiovascular
CI	confidence interval
CGM	continuous glucose monitoring
CTL	control
CRP	C-reactive protein
FMD	flow-mediated vasodilation
HbA1c	heamoglobin A1c
HCLF	high-carbohydrate, low-fat
HDL	high-density lipoprotein
hsCRP	high sensitivity CRP
HOMA IR	homeostatic model of insulin resistance
IL-6	interleukin-6
LCD	Low-carbohydrate diet
LCD	low-carbohydrate, high-fat
LDL	low-density lipoprotein
SD	means ± standard deviation
MUFAs	monounsaturated fatty acids
NID	nitroglycerine induced dilation
NAFLD	nonalcoholic fatty liver disease
NEFA	non-esterified fatty acid
RCT	randomised controlled trial
rQUICKI	revised Quantitative Insulin sensitivity Check Index
SFA	saturated fatty acids
TDD	traditional diabetic diet
TG	triacylglycerol
TNF-α	tumour necrosis factor-alpha
T2D	type 2 diabetes
